# Mentalization-based treatment for psychotic disorder: protocol of a randomized controlled trial

**DOI:** 10.1186/s12888-016-0902-x

**Published:** 2016-06-08

**Authors:** Jonas Weijers, Coriene ten Kate, Elisabeth Eurelings-Bontekoe, Wolfgang Viechtbauer, Rutger Rampaart, Anthony Bateman, Jean-Paul Selten

**Affiliations:** Rivierduinen Institute for Mental Health Care, Leiden, The Netherlands; Department of Psychiatry and Neuropsychology, South Limburg Mental Health Research and Teaching Network, EURON, Maastricht University, Maastricht, The Netherlands; Department of Clinical Psychology, Health and Neuropsychology, Leiden University, Leiden, The Netherlands; MBT Team, Anna Freud Centre, London, UK; Psychoanalysis unit, University College London, London, UK; Rivierduinen, GGZ Leiden, Sandifortdreef 19, room A426, 2333 ZZ Leiden, The Netherlands

**Keywords:** Mentalization, Treatment, Schizophrenia, Psychosis, Social functioning, Social cognition, Psychotherapy

## Abstract

**Background:**

Many patients with a non-affective psychotic disorder suffer from impairments in social functioning and social cognition. To target these impairments, mentalization-based treatment for psychotic disorder, a psychodynamic treatment rooted in attachment theory, has been developed. It is expected to improve social cognition, and thereby to improve social functioning. The treatment is further expected to increase quality of life and the awareness of having a mental disorder, and to reduce substance abuse, social stress reactivity, positive symptoms, negative, anxious and depressive symptoms.

**Methods/design:**

The study is a rater-blinded randomized controlled trial. Patients are offered 18 months of therapy and are randomly allocated to mentalization-based treatment for psychotic disorders or treatment as usual. Patients are recruited from outpatient departments of the Rivierduinen mental health institute, the Netherlands, and are aged 18 to 55 years and have been diagnosed with a non-affective psychotic disorder. Social functioning, the primary outcome variable, is measured with the social functioning scale. The administration of all tests and questionnaires takes approximately 22 hours. Mentalization-based treatment for psychotic disorders adds a total of 60 hours of group therapy and 15 hours of individual therapy to treatment as usual. No known health risks are involved in the study, though it is known that group dynamics can have adverse effects on a psychiatric disorder.

**Discussion:**

If Mentalization-based treatment for psychotic disorders proves to be effective, it could be a useful addition to treatment.

**Trial registration:**

Dutch Trial Register. NTR4747. Trial registration date 08-19-2014.

## Background and rationale

Non-affective psychotic disorders (NAPD) like schizophrenia are accountable for a substantial part of the total burden of disease, constituting the fifth and sixth leading cause of disability in the world, for men and women respectively [1]. A major contributor to this high level of disability is thought to be the decline in social functioning associated with NAPD. Patients with NAPD experience difficulty communicating [2], and tend to have poor social problem-solving skills [3]. These social deficits are predictive of poor vocational outcome [4] and poor quality of life [5]. It is surprising, therefore, that few treatments have been developed to effectively target them.

Social cognition – defined as “the ability to construct representations of the relation between oneself and others, and to use those representations flexibly to guide social behavior” [6] has been identified as one of the strongest predictors of social functioning in patients with NAPD [7]. Examples of social cognitive impairments in NAPD include difficulties recognizing emotions [8], empathizing [9] taking another person’s perspective [10] and understanding social hints [11]. In recent years just a few treatments, mostly rooted in cognitive/behavioral theory [12–14], have been developed that target social cognition. Results, are preliminary, but do suggest that impaired social cognitive deficits are targetable by psychosocial interventions [13–15].

Mentalization-based treatment for psychotic disorder (MBT-P) is based on a manualized psychodynamic treatment for Borderline Personality Disorder (BPD) [16]. Recent prospective studies suggest that psychodynamic therapy may improve global functioning in NAPD [17, 18], although randomized controlled trials should be conducted to substantiate this claim. Also, metacognitive psychotherapy [19] – which is closely related to MBT [20] – holds promise as a treatment for patients with schizophrenia [21]. MBT-P was developed to specifically improve social functioning by targeting the social cognitive process called ‘mentalizing’. Fonagy and colleagues [22] describe mentalizing as “the process by which we implicitly and explicitly interpret the actions of ourselves and others as meaningful on the basis of intentional mental states”. MBT adheres to a few important principles: (i) The therapist focuses on the current mental state of the patient to practice making representations of internal states; (ii) the therapist focuses on the present as opposed to the past; (iii) the therapist avoids talking about mental states that are not linked to subjectively felt reality; (iv) the therapist avoids talking about complex mental states; (v) the therapist focuses on recovering mentalizing, not creating insight [23].

This approach was found to reduce symptoms and interpersonal distress, and improve social functioning in patients with BPD [24]. Although BPD and NAPD may seem qualitatively different disorders, early views assumed borderline psychopathology occupied an area between neurosis and psychosis (e.g. Kernberg [25]) and could involve transient psychotic episodes. Since then, evidence has substantiated psychosis proneness in BPD. Psychotic symptoms, including hallucinatory experiences and delusions, occur regularly in patients with BPD, often persist over time, and are for a large part already present in early childhood [26]. In a recent study that included patients with either BPD or Schizophrenia, 17 % of participants met the criteria for both disorders [27]. Additionally, some NAPD and BPD patients share a tendency to excessively attribute incorrect intentions to others, or to “hypermentalize” [28–30]. Furthermore, disturbances in self-awareness and self-representation have been suggested to play an important role in both disorders [31]. Lastly, childhood trauma has been established as an important factor in the origins of both disorders [32]. Thus, as has been suggested earlier [20, 33], MBT may be a similarly suitable treatment for NAPD.

## Research aims and hypotheses

### Primary research aim

The primary aim of this study is to establish whether mentalization-based treatment for psychotic disorder (MBT-P) improves self-reported social functioning in patients with NAPD. We hypothesize that patients who receive MBT-P will show greater improvements in social functioning compared to patients who have had treatment as usual (TAU) only. We also expect that any difference observed will still be present at a 6 month follow-up.

### Secondary research aims

In addition to the self-reported level of social functioning, we will also examine global functioning as rated by researchers. Other outcome measures were chosen with previous research regarding MBT in mind. According to Fonagy and Bateman [34], MBT’s mechanism of change is improving patients’ mentalizing capacities. We therefore aim to establish whether MBT-P indeed increases mentalizing capacity, measuring several dimensions of social cognition, and whether this increase mediates a potential treatment effect. Furthermore, in earlier studies, Bateman and Fonagy [24] reported a reduction of anxious and depressive symptoms and of substance abuse. We expect similar results regarding NAPD patients. Given the strong relation between social functioning and quality of life, we assume that patients receiving MBT-P will report a higher quality of life. Additionally, based on previous research [35], we predict that improvement of social cognitive capacity will also lead to an increased awareness of having a mental disorder. Furthermore, as Bateman and Fonagy describe [34] that MBT was designed to improve emotion regulation in situations of attachment related (i.e., social) stress. Based on this, we assert that patients will have less aversive emotional reactions to situations of social stress as a result of MBT-P. If MBT-P can reduce patients’ emotional reactivity to social stress, they may also become less prone to develop positive psychotic symptoms, as social stress reactivity may be an affective pathway to psychosis [36]. Lastly, because social functioning and mentalizing ability have been found to be strongly related to negative symptoms, we will examine whether MBT-P reduces negative symptoms [37–39].

### Covariates

Certain potential effect modifiers will be taken into account. First, adverse childhood experiences such as neglect or physical, psychological, and sexual abuse have been associated with social dysfunction later in life [40]. Second, personality organization (PO) has been shown to impact psychotherapy treatment response [41] and the level of social cognition [42]. Five levels of personality organization (PO) are identified, each characterized by different levels of anxiety tolerance and different capacities for impulse inhibition or ‘control’: Neurotic PO (good anxiety tolerance, over-control); Borderline PO (moderate to poor anxiety tolerance, differing levels of control); Narcissistic Borderline PO (good anxiety tolerance, undercontrol); Latent Psychotic PO (moderate anxiety tolerance, weak control); and Manifest Psychotic PO (poor anxiety tolerance, weak control). Third, the awareness of bodily sensations has been regarded as the first level of emotional awareness [43], which is an essential part of social cognition [44]. Psychopathology usually is accompanied by (vague) physical complaints, called “somatization”. A failure to report physical complaints while suffering (severe) psychopathology may be indicative of an absence of bodily awareness and therefore an impaired social cognitive capacity. Fourth, medication use can affect social functioning in patients with NAPD, therefore the type of medication is registered and adherence to medication is measured. Fifth, MBT attendance is taken into account, because it is expected that those who attend more sessions will profit more. Sixth, baseline measurements of the outcome variables will be accounted for. Seventh, the duration of illness, because of its negative impact on functioning; and eighth, adherence to the MBT model by therapists, because it is likely to influence treatment outcome.

## Methods/design

### Trial design/setting

This study is a rater-blinded, randomized controlled trial. Patients referred to outpatient sites of the Rivierduinen mental health institute are randomly assigned to Treatment as Usual (TAU) plus MBT-P or TAU only. The Rivierduinen mental health institute provides in- and outpatient treatment to thousands of patients with psychiatric disorders in the Dutch province of South-Holland (e.g., Leiden, Gouda). The investigator and patients are aware of treatment allocation, but all measurements are performed by researchers blind to treatment allocation. Social functioning at baseline (t0) and after treatment (t2) will be compared for both treatment conditions. The patients are blind to this primary aim.

### Participants

Participants in the study are patients with NAPD (DSM-IV criteria [34]): schizophrenia, schizophreniform, or schizoaffective disorder (295.x), delusional disorder (297.1), brief psychotic disorder (298.8), or psychotic disorder not otherwise specified (298.9). At least 80 participants will be included from the Rivierduinen mental health institute.

Inclusion criteria are:

- At least 6 months of prior treatment.
- No more than 10 years of treatment for NAPD.
- Between 18 and 55 years of age.

Exclusion criteria are:

- Intellectual disability and/or illiteracy.
- A lack of mastery of the Dutch language.
- Substance abuse to such an extent that it necessitates inpatient detoxification. After detoxification the patient is still eligible for participation in the study. Patients cannot participate in a session while under the influence of drugs.

### Sample size calculation

The estimated effect size of the current study is based on two previous studies. A study examining the effect of MBT on BPD [35] showed moderate to large reductions of problems relating to interpersonal distress (d = 0.95; 95 %; CI: 0.59–1.30) and social functioning (d = 0.72; 95 % CI 0.37–1.06). A study concerning Social Cognition and Interaction Training (SCIT) – a treatment for patients with schizophrenia that shares many elements with MBT – showed large effects on social engagement (d = 1.77) and interpersonal communication (d = 1.57) on the Social Functioning Scale [36]. Based on these results, we expect to find a moderate to large effect of MBT-P on social cognition (i.e., a Cohen’s d of at least 0.7).

To calculate the required sample size, G*Power [37] was used. To obtain a significant difference with power equal to .80 with an independent samples t-test (for a true effect size of .7 and alpha = .05), 68 participants are needed. However, because repeated measurements (baseline and post-treatment) increase the power – depending on the test-retest reliability of the outcome measure – a smaller sample size is required. A formula has been devised [38] to account for the increased power when using multiple measurements: n_repeated measures_ = (1-ρ^2^)*n_t-test_; where ρ is the test-retest reliability of the scale used. Previous research has shown that the test-retest reliability of the social functioning scale after two and a half years is ρ = .40 [39]. Thus we need: (1-0.4^2^)*68 ≈ 58 participants to have 80 % power to find a significant difference.

It is difficult to predict the amount of drop-out in the study. In an unpublished pilot study, conducted at the Rivierduinen mental health institute, the drop-out rate of MBT-P within a period of 1 year was 10 %. Since the current RCT combines treatment and measurements, we estimate that the drop-out rate will be higher. Therefore, to be sure, we will recruit at least 40 (TAU) + 40 (TAU plus MBT-P) = 80 individuals. Even if 25 % of those initial patients (i.e., 20) drop out, there will be 60 patients left to detect an effect. Because patients in the MBT-P condition receive therapy in groups of up to eight persons, five groups will be formed in order to recruit 40 patients in the MBT-P condition.

### Procedure

Psychiatrists, psychologists, or psychiatric nurses will check their caseload for patients who meet the requirements for participation and ascertain whether they are interested in participating. If this is the case, the researcher will provide an information letter. The patient will be given 7 days to read the letter and to decide whether he/she wants to participate. At a second appointment, the researcher will check whether the patient has understood the information in the letter. Subsequently, both parties sign an informed consent form in twofold. Randomization is performed by an independent external agency.

## Measurements and instruments

There are four moments of assessment over the course of 2 years. The baseline assessment takes place before MBT-P is started. Furthermore, there will be an assessment after 9 months (halfway MBT-P), after 18 months (directly after MBT-P has ended), and after 24 months (6 months after MBT-P has ended). See Table 1 for the specific instruments that are used at each assessment.

**Table 1:** Overview of the different instruments at each measurement moment

	Intake	T0: Baseline measurement (0 months)	T1: Halfway Measurement (9 months)	T3: End of Treatment (18 months)	T4: Follow-up (24 months)
CASH	X				
SFS		X		X	X
TAT		X		X	X
ESM		X	X	X	X
HT		X		X	X
MANSA		X		X	X
MAQ		X	X	X	X
DSFM		X			
PANSS		X		X	X
GDQ		X			
CECA		X			
GAF		X		X	X

### Diagnosis

All patients are diagnosed according to DSM-IV criteria [45] by a psychiatrist. Prior to participation, this diagnosis will also be verified using the Comprehensive Assessment of Symptoms and History (CASH). The CASH is a semi-structured interview that documents signs, symptoms, and history of psychotic, manic and depressive syndromes as well as substance abuse. The instrument has been extensively tested concerning interrater reliability, test-retest reliability and validity [46].

### Social functioning

Social functioning is measured using the Social Functioning Scale (SFS), a self-report questionnaire. The SFS has been found to be reliable, valid, sensitive, and responsive to change [47]. The scale contains seven dimensions of global social functioning that are especially pertinent to patients suffering from psychotic disorders. The dimensions are: social withdrawal, interpersonal communication, independence (competence), independence (performance), recreational activities, social activities, and employment.

Secondly, using the modified GAF scale [48], global functioning will be assessed. The modified GAF scale is a clinician or researcher-rated instrument, which makes it a good addition to the self-reported SFS. It has more detailed criteria and a more structured scoring system than the original GAF, which is underscored by a high interrater reliability.

### Social cognitive capacity

It has been pointed out that there is to date no agreement on the assessment of social cognition, but there is a broad consensus that it is a multifaceted construct [49]. In the current study, social cognition is therefore assessed with two instruments that measure different aspects of social cognition: the Thematic Apperception Test (TAT) and the hinting task (HT). The TAT [50], scored with the Social Cognition and Object Relations System (SCORS) [51], is used to assess four dimensions of social cognition: complexity of representations of people and understanding of social causality, which comprise cognitive aspects of social cognition, and the affect-tone of relationships and the capacity for emotional investment, comprising affective aspects of social cognition. Each dimension is scored on a 5-point scale, with higher scores representing higher social cognitive functioning in that dimension. Six pictures of the TAT are used. TAT responses are recorded and transcribed verbatim. TAT responses, when analyzed with the SCORS, have been found to be a valid and reliable way to measure social cognition and object relations [52, 53]. According to Luyten and colleagues [54], the TAT is one of the few tests that takes almost all aspects of mentalization into account, including affective and cognitive aspects.

The HT [37] is used to measure the ability to infer intentions from others, or ‘Theory of Mind’ (ToM). Patients read extracts that describe an interaction between two characters in which one character says something with an implicit message. If the patient infers the implicit message correctly, two points are scored. If a hint is needed, a score of 1 is given. When the answer is incorrect or the participant does not know, 0 points are scored. The test comprise ten short passages. The Hinting Task has very recently been reviewed [55] regarding test-retest reliability, utility as a repeated measure, relation to functional outcome, and internal consistency and was one of two tests shown with strong psychometric properties across all evaluation criteria.

### Social stress reactivity

Emotional reactivity to stress in social situations is measured with an electronic diary using the ‘Experience Sampling Method’ (ESM) [56]. ESM is a repeated self-assessment technique with great ecological validity. Participants carry around an ESM device, which facilitates the monitoring of daily life experiences and behavior. Ten times daily on five consecutive days, it generates an audible signal (beep) at unpredictable moments of the day which participants answer by using the touch screen of the device. Participants are asked whether they are alone or in company of others. Then, both the level of social stress and negative/positive affect are assessed. Social stress is measured with items such as: “I would rather be alone” and “I like the present company” (reverse coded). Negative affect is the averaged score of the mood items “anxious”, “lonely”, “insecure”, “irritated”, “down”, “guilty”, and “gloomy”. Positive affect will be measured with the items “happy”, “satisfied”, “cheerful”, “relaxed”, and “enthusiastic”. All items are scored on a 7-point Likert Scale.

### Quality of life

Using the Manchester Short Assessment of quality of life (MANSA) [57], changes in overall quality of life are measured. The MANSA is a 16-item, 7-point Likert-scale self-rating instrument.

### Psychotic symptom severity

Interviewers use the Positive and Negative Syndrome Scale (PANSS) [58] to assess positive (subscale P), negative (subscale N), anxious (item G2), and depressive symptoms (item G4). The PANSS is a 30-item, 7-point Likert-scale rating instrument developed for the assessment of phenomena associated with schizophrenia. A Dutch version is used [59].

Additionally, the ESM diary is used to measure momentary psychotic experiences. Seven ESM items are used: ‘I feel suspicious’, ‘I am afraid of losing control’, ‘I feel that others don’t like me’, ‘I feel that others want to hurt me’, ‘My thoughts are influenced by other people’, ‘I feel unreal’, and ‘I hear voices’.

### Awareness of having a mental disorder

The PANSS (item G12) is used to assess awareness of having a mental disorder.

### Substance abuse

Patients are asked to report substance use on the ESM device at each beep using categorical questions. Patients will report whether they have used any substance since the last beep, including: (1) caffeine, (2) nicotine, (3) medication, (4) alcohol, (5) cannabis, (6) other drugs, or (7) none.

### Personality organization/somatization of psychopathology

Assessment of personality organization and the tendency to somatize severe psychopathology is conducted using theory driven profiles of the Dutch short Form of the MMPI (DSFM) [60], an 83-item self-assessment questionnaire. The DSFM measures personality traits on 5 scales: Extraversion, Psychopathology, Shyness, Somatization, and Negativism. Using the theory driven profile approach to the DSFM [61–65], five levels of Personality Organization (PO) are distinguished: Neurotic PO, Borderline PO, Narcissistic Borderline PO, Latent Psychotic PO and Manifest Psychotic PO. To measure bodily awareness, the DSFM “Somatization” subscale (20 items) will be used. This subscale measures the amount and degree of experienced bodily symptoms, and hence, the ability to subjectively report, and be aware of bodily sensations. As described [65] affect regulation through somatization will be expressed as the relative position of scores on the subscale somatization to that on the severe psychopathology subscale.

### Childhood trauma

The Childhood Experience of Care and Abuse (CECA) [66] is a semi-structured interview that aims to assess details and the time-sequence of traumatic childhood experiences. It assesses lack of care (neglect, antipathy), physical abuse, sexual abuse, and psychological abuse.

### Adherence to drug treatment

Each patient’s medical record is consulted to ascertain the pharmacotherapy prescribed at t0, t1, t2, and t3. Adherence to the prescribed medication is measured with the Medication Adherence Questionnaire (MAQ) [67].

### Adherence to the MBT-model

Adherence to the MBT model by therapists is rated by an experienced MBT therapist according to the MBT adherence and competence scale [68], using footage of therapy sessions. Therapists are judged on 17 items that characterize proper MBT treatment.

### Duration of illness

Assessed using the CASH, refers to the period since first psychosis.

### General demographics

The general demographic questionnaire (GDQ) is a standard instrument used in all ESM studies conducted at Maastricht University. It documents treatment history, socio-economic status, educational level, and urbanicity of the place of residence.

### Treatment

#### TAU

All patients in the current study receive a multifaceted treatment based on the ‘Functional Assertive Community Treatment’ (FACT) model. FACT teams consist of psychiatric nurses, welfare workers, psychologists, and at least one psychiatrist. The intervention consists of pharmacotherapy, case-management, psycho-education, and in some cases cognitive behavioral therapy (CBT). CBT interventions for psychosis usually take around 20 sessions and are based on the work of van der Gaag [69, 70]. Whether or not patients have received CBT, and how many sessions, will be registered and taken into account.

Furthermore, all patients will receive supportive-structuring therapy. Sessions focus on problems patients may encounter in their social network, work, daily activities, or medication adherence. Patients meet with a mental health professional for an average of 30 min every 2 weeks, with a minimum of 1 meeting of 30 min per month, over 18 months. The total number of individual sessions is estimated to be around 30 (15 h of individual therapy). Adherence to treatment sessions is monitored by registering patients’ presence in the sessions.

#### MBT-P

Patients in the TAU plus MBT-P condition receive the same treatment mentioned above in combination with individual and group MBT-P. The key elements of MBT, shortly described below, provided the basis for MBT-P [16].

#### Therapeutic stance

MBT is characterized by the ‘not knowing stance’ in which the therapist admits to not knowing what the patient experiences. By actively asking questions the therapist cultivates an attitude of sincere interest in the patient. This gives the patient the experience of being ‘kept in mind’ by someone, but also stimulates curiosity in the patient towards his own mental states.

#### Interventions

When applying interventions, it should always be kept in mind that arousal tends to diminish the capacity to mentalize. Four stages of intervention can be used in a step-wise manner, depending on the level of arousal. At the first level, interventions are aimed at down-regulating arousal in the patient. These include empathic validation of the patient’s feelings and complimenting good mentalizing. At the second stage the therapist asks for clarification of a situation or elaboration on the patient’s feelings and thoughts. Often the therapist stops or rewinds the patient’s narrative to investigate an aspect of the dialogue. This stage is about making implicit mentalizing explicit. Often details are investigated that seem to affect the patient, or should affect the patient, but do not. This then leads to the next stage, called ‘mentalized affectivity’, which is the activity of reflecting on emotions, while simultaneously experiencing them. It is considered to be a crucial aspect of emotion regulation. The explicit mentalizing of a primary affective experience gives the patient the opportunity to express (or inhibit) emotions in a non-automatic manner. At the last stage, the relationship between therapist and patient or between patients is mentalized. In this stage, both patient and therapist reflect on and share their affective experience to become aware how their relationship is affecting them. Care should be taken applying this stage of intervention, as it requires a robust level of mentalizing.

#### Duration and dose

Compared to original MBT, the length of MBT-P, has remained unchanged: 18 months. However, the frequency and length of sessions has been reduced. In our experience, based on a pilot MBT-P intervention, NAPD is associated with more severe mentalizing deficits than BPD, as has also been suggested elsewhere [71]. Given, the danger of overwhelming NAPD patients with mentalizing interventions, group therapy is limited to weekly 1-hour sessions, while individual therapy takes place in biweekly half-hour sessions. We feel this approach is justified by the low drop-out rate of 10 % in the pilot intervention.

#### Psycho-education

MBT-P starts with two sessions of psycho-education, in which patients are told about the key aspects of MBT, including the meaning of mentalizing and its sensitivity to arousal.

#### Individual therapy

Individual therapy provides an opportunity for intensive practice in mentalizing. The focus is on establishing a secure relationship that acts as a safe base from which failures in mentalizing can be explored. Treatment goals on the basis of five problem areas are developed and routinely reviewed with the patient, including: commitment to treatment, psychiatric symptoms, social interaction/relationships, destructive behavior, and community functioning.

#### Group therapy

Since patients with NAPD tend to experience a great deal of stress in social situations, group therapy provides an opportunity to practice mentalizing in a stress-evoking setting. Group therapy can also generate a sense of belonging and attachment that help foster mentalizing. The group size is a maximum of eight patients and there will be one MBT-P therapist and one co-therapist present at each session.

#### Therapists

Group therapists are experienced and registered MBT therapists. Individual therapists are mental health professionals (psychologists, psychiatric nurses or psychiatrists) who receive training to become MBT-P therapists. Supervision is provided in weekly sessions of up to 1h, during which therapists reflect on their interventions and whether they are faithful to the MBT model.

### Statistical analysis

#### Main effect

The main purpose of the statistical analysis is to compare the overall effect of treatment condition on social functioning. For this, an ANCOVA will be used with treatment condition as between-subjects variable, post-treatment social functioning as dependent variable, and baseline social functioning as a covariate. Other potential covariates include childhood trauma, level of PO, somatization of psychopathology, medication use, attendance of MBT-P sessions, duration of illness and adherence to the MBT-model by therapists. Similar analyses will be conducted for the secondary outcome measures (social cognition, quality of life, awareness of having a mental disorder, anxious, depressive, negative and positive symptoms and substance abuse).

Since drop-out will undoubtedly result in missing data, the possibility of attrition-bias is a cause of concern [72]. For example, it is conceivable that those who fare the worst in therapy tend to drop out, thus creating a biased sample. Following the advice of Altman [73], the analyses will therefore be conducted on the basis of ‘intention to treat’ (ITT), meaning that they will include all patients who sign up, regardless of actual participation in the entire program. Missing data will be handled by means of multiple imputation (MI) [74]. In the current case missingness is most likely to be caused by participants dropping out of their respective treatment programs. Since certain variables have been found to influence drop-out rates in patients with NAPD, we cannot assume that the data are missing at random. A review [75] identified a lack of insight, poor social functioning, positive symptoms, young age, male gender, a history of drug abuse, and unemployment as key predictors of treatment program drop out. Thus in the current study, these variables will be used to predict missingness. Additionally, treatment condition will be used as a predictor as well, because the time investment differs between conditions. For each analysis, a total of 5 imputed datasets will be created using a fully conditional Markov chain Monte Carlo (MCMC) approach, which will be combined using standard procedures [76].

#### Mediation

**Fig. 1:**
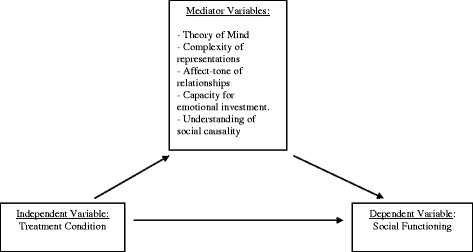
Theoretical model of the effect of treatment condition on social functioning, mediated by social cognition

We also aim to examine whether changes in various social cognitive dimensions mediate the potential increase in social functioning. In order to test this mediational model, we will carry out a multi-mediator analysis [77]. This allows for a parallel testing of the indirect effects of several social cognitive dimensions, namely: theory of mind, complexity of representations, affect-tone of relationships, capacity for emotional investment and understanding of social causality (Fig. 1).

#### Multilevel analysis

Social stress reactivity is measured by ESM. This method generates data with a multilevel structure because there are multiple measurements per day for 5 days at a time for each patient. Since observations within patients tend to be more similar than observations from different patients, they are not independent. This necessitates a different (i.e., multilevel) analysis than the one used for the other outcome measures. Similar to Myin-Germeys and colleagues [78], differences in social stress reactivity between groups will be analyzed using mixed-effects regression models with treatment condition, the amount of stress during social situations, and their interaction term as independent variables, and positive and negative affect as dependent variables. The model will include random intercepts and random slopes for the stress predictor at the patient level, which allows for differences in overall levels of positive and negative affect across patients and for differences in the strength of the relationship between stress and these outcomes.

## Discussion

There is evidence that MBT improves, among others, social functioning and interpersonal distress in patients with BPD (e.g. [24, 79]) and that these effects remain at follow-up [80]. Given the similarities in both origins and symptoms of BPD and NAPD, it has previously been suggested that MBT could be a useful treatment for NAPD [20, 33]. This randomized controlled trial, will be the first to examine the effectiveness of MBT as an adjunct therapy for patients with NAPD. Furthermore, we aim to determine a possible mechanism of change by examining social cognitive capacity as a possible mediator. By measuring social cognitive capacity with two instruments, covering a total of five dimensions, we mean to do justice to its complexity.

## Abbreviations

(Z)MLK, Abbreviation for the Dutch term ‘(zeer) moeilijk lerende kinderen’ meaning: children with (severe) learning disabilities; BPD, borderline personality disorder; CASH, comprehensive assesment of symptoms and history; CBT, cognitive behavioral therapy; CECA, childhood experiences of care and abuse; DSFM, Dutch short form of the Minnesota Multiphasic personality inventory; ESM, experience sampling method; GAF, global assessment of functioning; GDQ, general demographic questionnaire; HT, hinting task; ITT, Intention to treat; MANSA, Manchester short assessment of quality of life; MAQ, medication adherence questionnaire; MBT, mentalization-based treatment (for borderline personality disorder); MBT-P, mentalization based treatment for psychotic disorders; MCMC, markov chain monte carlo; MI, multiple imputation; MMRM, mixed model for repeated measures; MREC/METC, medical research ethics committee (MREC); in Dutch: medisch ethische toetsing commissie (METC); NAPD, nonaffective psychotic disorder; PANSS, positive and negative syndrome scale; PO, personality organization; SCORS, social cognition and object relations system; SFS, social functioning scale; TAT, thematic apperception test; TAU, treatment as usual
